# Affinity Purification of *O*-Acetylserine(thiol)lyase from *Chlorella sorokiniana* by Recombinant Proteins from *Arabidopsis thaliana*

**DOI:** 10.3390/metabo4030629

**Published:** 2014-08-04

**Authors:** Giovanna Salbitani, Markus Wirtz, Rüdiger Hell, Simona Carfagna

**Affiliations:** 1 Dipartimento di Biologia, Università di Napoli Federico II, Via Foria 223, I-80139 Napoli, Italy; E-Mail: simcarfa@unina.it; 2 Centre for Organismal Studies, University of Heidelberg, Im Neuenheimer Feld 360, D-69120 Heidelberg, Germany; E-Mails: markus.wirtz@cos.uni-heidelberg.de (M.W.); ruediger.hell@cos.uni-heidelberg.de (R.H.)

**Keywords:** amino acids, cysteine synthase complex, microalgae, nutrition, serine acetyltransferase, sulphur metabolism

## Abstract

In the unicellular green alga *Chlorella sorokiniana* (211/8 k), the protein *O*-acetylserine(thiol)lyase (OASTL), representing the key-enzyme in the biosynthetic cysteine pathway, was isolated and purified to apparent homogeneity. The purification was carried out in cells grown in the presence of all nutrients or in sulphate (S) deprived cells. After 24 h of S-starvation, a 17-fold increase in the specific activity of OASTL was measured. In order to enable the identification of OASTL proteins from non-model organisms such as *C. sorokiniana*, the recombinant his-tagged SAT5 protein from *Arabidopsis thaliana* was immobilized by metal chelate chromatography. OASTL proteins from *C. sorokiniana* were affinity purified in one step and activities were enhanced 29- and 41-fold, from S-sufficient and S-starved (24 h) cells, respectively. The successful application of SAT/OASTL interaction for purification confirms for the first time the existence of the cysteine synthase complexes in microalgae. The purified proteins have apparent molecular masses between 32–34 kDa and are thus slightly larger compared to those found in other vascular plants. The enhanced OASTL activity in S-starved cells can be attributed to increased amounts of plastidic and the emergence of cytosolic OASTL isoforms. The results provide proof-of-concept for the biochemical analysis of the cysteine synthase complex in diverse microalgal species.

## 1. Introduction

Sulphur is an essential nutrient for plant growth and development; it represents the least abundant essential macronutrient in plants [1,2]. In all photosynthetic organisms, including algae, inorganic sulphur is absorbed as sulphate which is usually easily accessible in soil and aquatic ecosystems [3]. The allocation of sulphate around the plant and movement among the cell compartments is facilitated by specific sulphate transporters (SULTR) [4]. In *Arabidopsis thaliana*, the family of sulphate transporters is encoded by 14 genes [5]. In *Chlamydomonas reinhardtii*, two distinct sulphate transport systems are localized in the plasma membrane and a bacterial type sulphate permease in the chloroplast envelope, respectively [6,7,8].

In plant cells, sulphur assimilation is realized in four consecutive stages: S uptake, S activation, reduction of activated S and cysteine biosynthesis. Today, the sulphur assimilation pathway is most widely studied in vascular plants compared to algae. However, microalgae in their marine environments underwent an evolutionary history of changing sulphate concentrations, while today facing a constantly high sulphate concentration of about 30 mM [9,10]. In contrast, fresh water algae are exposed to much lower strongly changing sulphate concentrations in their environment, leading to S-deficiency inducible acquisition and uptake systems [3,8]. The subsequent steps of intracellular activation and reduction proceed by the same steps in land plants and algae [3,4].

Cysteine (Cys) is the first amino acid that is produced by the sulphur assimilation pathway and the starting point for production of methionine, glutathione and a variety of other sulphur-containing metabolites [2]. Cys biosynthesis is the last step of sulphur assimilation and proceeds by two interconnected and consecutive reactions catalyzed by two enzymes: serine acetyltransferase (SAT, EC 2.3.1.30) and o-acetylserine(thiol)lyase (OASTL, EC 4.2.99.8). SAT acetylates L-serine from acetyl-CoA to form o*-*acetylserine (OAS), the activated form of serine and substrate of the OASTL reaction; OAS subsequently condensates with sulphide in the reaction catalyzed by OASTL to form Cys. In vascular plants both enzymes are localized in the chloroplasts, mitochondria and cytosol with different functions for Cys synthesis [11,12,13]. Non-vascular plants such as *Physcomitrella* apparently synthesize cysteine only in the chloroplast and cytosol but not in mitochondria [14]. In the unicellular green alga *Chlorella sorokiniana* two OASTL isoforms, chloroplastic and cytosolic, were suggested, among which the cytosolic was induced under S-deprivation [15]. The genome of the closely related microalga *Chlamydomonas reinhardtii* encodes for several isoforms of SAT and OASTL proteins that are all presumed to be localized in the chloroplast [16]. A similar genomic organisation has been observed for the diatom species *Thalassiosira pseudonana* [17] and *Phaeodactylum tricornutum* [18]. In *Chlamydomonas* the isoforms SAT1 and OASTL4 are induced during S-starvation [19] and enhanced OASTL activity has been reported [20]. These observations indicate a regulatory difference, since in vascular plants, genes encoding SAT respond only weakly and those encoding OASTLs are constantly expressed during S-deficiency [12]. The tasks and relative contributions of the different sites of Cys synthesis in algae are currently not known, making more knowledge on their regulation with respect to sulphur metabolism desirable.

In vascular plants, protein-protein interactions between SAT and OASTL lead to the formation of the cysteine synthase complex (CSC) which plays an essential regulatory role in Cys biosynthesis [12]. For soybean, Kumaran and co-workers [21] used analytical ultracentrifugation and size-exclusion chromatography analysis to develop a model of a CSC containing a SAT trimer associated with three OASTL dimers having the molecular weight of 310 kDa. In contrast, the analyses by Wirtz *et al.* [22] using similar methods to analyze soybean and *Arabidopsis* CSCs, confirmed the originally proposed quaternary for the bacterial CSC [17]. According to the latter, a hexameric SAT interacts with two OASTL dimers. This structure was further supported by kinetic docking modelling [23]. A regulatory function of the CSC for the rate of Cys synthesis has been suggested which is based on the association/dissociation of the two enzymes, SAT and OASTL, triggered by the availability of OAS and sulphide [24]. In plants and also in bacteria, OASTL is catalytically inactive in the CSC but becomes fully active upon dissociation from the complex realized by OAS [22]. The rapid and stable formation of CSC would allow production of OAS to maintain intracellular Cys levels during high demand conditions. In addition, the feedback sensitivity of SAT to Cys is considerably lower in the CSC as compared to free SAT, allowing for elevated OAS production and subsequent Cys synthesis by free OASTL [21,22].

Despite the many studies concerning the synthesis of Cys in vascular plants [12,25], only some dealt with the same subject on algae [15,20]. Today, little is known about the occurrence of the CSC in the microalgae, the intracellular localization of SAT and OASTL enzymes and the regulation of cysteine synthesis.

Furthermore, the utilization of unicellular algae as a model system to study enzymes involved in plant nutrition is generally advantageous because the metabolism responds uniformly to nutrient supply that each cell uptakes from the medium. To investigate the occurrence of the regulatory CSC in microalgae, we considered as organism of study *Chlorella sorokiniana* (strain 211/8 k), a single cell, fresh water green algae (*Chlorophyta*), that represents a suitable and long-established experimental system to study phenomena deriving in plant cells from sulphur starvation or S-supply. In addition, *Chlorella sorokiniana* reproduces faster (about 6 h) [15,26], with respect to the algal model organism *Chlamydomonas reinhardtii* [26], giving the opportunity to study cellular metabolic processes in a short span of time. Very importantly, in recent years *Chlorella* species have become the most widely used microalgal strains for biotechnology applications and biomass production [27,28,29].

In this study we investigated the presence of the CSC, the enzymatic activity of OASTL under S-deprivation and the molecular mass of OASTL proteins in *Chlorella*. The rationale for these experiments was the reported uniquely altered amounts and activities of OASTL during S-starvation as opposed to the vascular plant systems [12,19]. This likely reflects the different habitats and speed of environmental changes which make these responses necessary. The existence of the cysteine synthase complex also in microalgae would allow us to analyze the potentially different regulatory mechanisms between the well-investigated vascular plant and ill investigated algal systems as well as differences between algal taxa and algae from different habitats. To this end, OASTL proteins from the alga were purified to apparent homogeneity using recombinant SAT (AtSAT5) from *Arabidopsis thaliana* as affinity anchor.

## 2. Results and Discussion

### 2.1. OASTLs Purification and CSC

**Figure 1:**
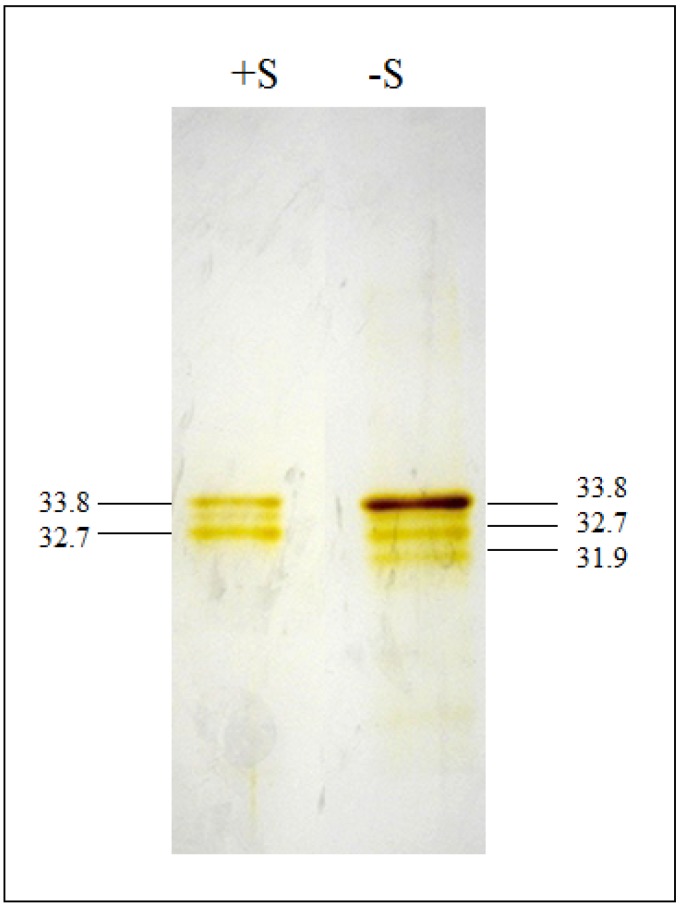
Silver-stained SDS-PAGE analysis of purified *O*-acetylserine(thiol)lyase (OASTL) proteins. +S: purified OASTL proteins from S-sufficient cells; −S: purified OASTL proteins from S-starved cells for 24 h. Proteins loaded in each lane were 80 μg. The apparent molecular mass of 33.8 kDa refers to the plastid OASTL and 31.9 kDa to the cytosolic OASTL.

The enzymes OASTL were purified from S-sufficient (+S) and S-starved for 24 h (−S) algal cultures by SAT-OASTL affinity chromatography. The 24 h starvation period was based on earlier observations and aimed at the characterization of the biological system with respect to the change in cysteine synthesizing enzymes during acclimation. The cytosolic AtSAT5 isoform from *Arabidopsis* was *N*-terminaly fused with a histidin tag, expressed in *E. coli* and immobilized on a Hi-trap column [22]. The immobilized AtSAT5 bound *Chlorella* OASTL proteins from crude cell extracts (Figure 1). The identity of the eluted proteins is based on the following evidence: (1) the eluted fraction contained strongly enriched OASTL enzyme activity; (2) the elution was carried out with OAS, the specific effector of CSC dissociation; (3) the proteins were purified to apparent homogeneity based on highly sensitive silver staining. OAS is known to function exclusively in Cys synthesis in plants and bacteria [30,31].

OASTL was enriched 29 times from S-sufficient cells (Table 1) and 41 times from S-starved cells (Table 2) based on specific activity. According to OASTL activity in crude extracts, the efficiency was very high with yields of 77% and 58%, respectively. This shows that *Chlorella* OASTL and *Arabidopsis* readily interact *in vitro* and form a heterologous CSC, strongly suggesting interaction of endogenous SAT and OASTL in *Chlorella*. The finding also corroborates the ability of SATs and OASTLs to form hybrid complexes between proteins from different cellular compartments and even plant species [24,30]. The decisive site for binding of plant and bacterial SATs to their OASTL counterparts consists in the very four *C*-terminal amino acids and in particular in the presence of a terminal isoleucine [21,22]. Indeed, the two SAT isoforms present in *Chlamydomonas* have *C*-termini with significant conservation to those of land plants [16], possibly providing a structural explanation also for the SAT-OASTL interaction in *Chlorella*. This result implies the applicability of this straightforward purification approach to non-model algal taxa whose genomes have not yet been sequenced and thus to functional studies of their mechanisms of cysteine synthesis.

**Table 1:** Representative purification table of OASTL enzymes from *Chlorella sorokiniana* S-sufficient cells (+S).

*Fraction (+S)*	*Total Protein (mg)*	*OASTL Total Activity (U·mL^−1^)*	*OASTL Specific Activity (U·mg^−1^ prot)*	*Yield (%)*	*Purification Level*
***Crude extract***	4.8	24.85	5.2	100	1
***Elution***	0.13	19.2	149	77	29

**Table 2:** Representative purification table of OASTL enzymes from *Chlorella sorokiniana* S-starved cells for 24 h (−S).

*Fraction (–S)*	*Total protein (mg)*	*OASTL total activity (U·mL^−1^)*	*OASTL specific activity (U·mg^−1^ prot)*	*Yield (%)*	*Purification level*
***Crude extract***	1.4	35	25	100	1
***Elution***	0.02	20.4	1020	58	41

Notably, the specific activity of the eluted and purified OASTL fraction in S-starved cells from *Chlorella* was approximately three times higher compared to the one prepared from leaf extracts of *Arabidopsis* using homologous SAT affinity chromatography [32]. Since the yields and purities in the preparations from both sources were similar, this suggests more active OASTL protein species in the alga compared to the land plant in addition to the inducibility of the total activity under S-starvation [20]. The higher specific activity of OASTL and the inducibility under S-deprivation are marked differences of the algal system as compared to vascular plants, possibly reflecting the requirement of a more pronounced acclimation response to nutrient changes (here sulphate) in the environment.

The purity of OASTL protein was examined by SDS-PAGE (Figure 1). The purification in one step was highly specific and achieved apparent homogeneity despite the evolutionary distance and concomitant primary sequence diversion between algal and *Arabidopsis* OASTL with respect to binding to SAT. S-sufficient and S-starved purified extracts showed different protein patterns. Purified OASTL proteins from S-sufficient cells migrated as two sharp bands corresponding to molecular masses of approximately 33.8 and 32.7 kDa. The 33.8 kDa band is in good agreement with the size of the chloroplast OASTL isoform from *Chlorella* that had been identified by immunoblotting [15], while the much weaker band of 32.7 kDa is likely a product of its degradation. In S-starved cells the same bands were visible plus an additional lower band with an estimated molecular mass of 31.9 kDa. This band corresponds well to the size of the cytosolic OASTL isoform [15]. Thus, the apparent molecular masses found here were slightly lower than in *Arabidopsis thaliana* [12]. The intensity of the upper band (33.8 kDa) was clearly increased and the lowest (31.9 kDa) band was comparatively weak. Remarkably the assumed degradation product (32.7 kDa) did not change in abundance, possibly suggesting that the degradation rate was not concomitantly increased under S-deprivation conditions. Thus, S-starvation resulted in the increase of the larger chloroplastic OASTL isoform and the induction of a new and smaller cytosolic isoform.

### 2.2. OASTL Activities

**Figure 2:**
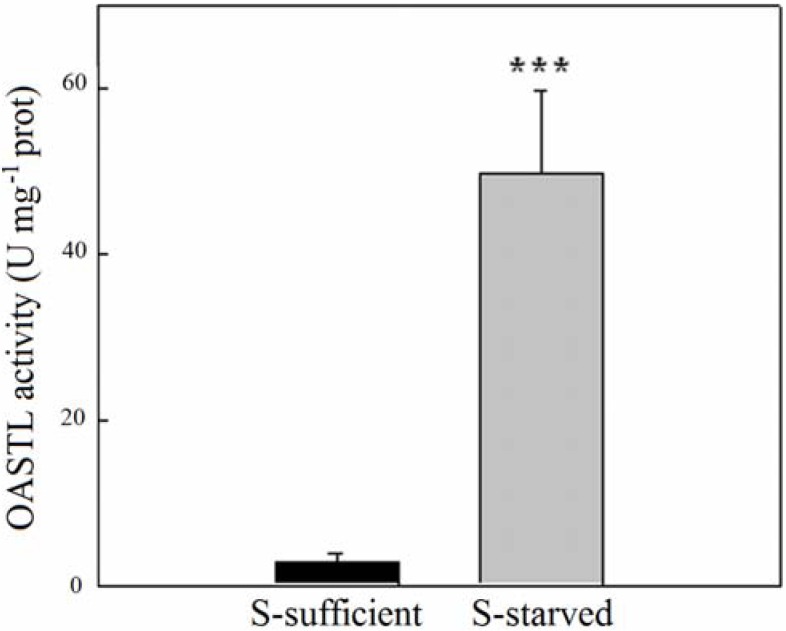
Effects of S-starvation on OASTL activity in cells of *Chlorella sorokiniana*. The black bar chart represents S-sufficient cells, grey bar chart represents S-starved cells for 24 h. The values reported are means ± SE (*n* = 3) of equally extracted and treated samples in S-sufficient and S-starved cells. Significant differences, using one-way ANOVA, were analyzed in S-starved cells with respect to S-sufficient cells (*p* < 0.001, ***).

In *Chlorella sorokiniana*, the removal of S from the culture medium caused a strong increase in OASTL activity; these were measured spectrophotometrically in S-sufficient cells and in cells S-starved for 24 h. Compared to S-sufficient cells, S-starvation for 24 h caused a strong increase of OASTL specific activity. In fact, the OASTL activity in S-sufficient algae was 2.8 ± 1 U·mg^−1^ protein while in S-starved cells was increased about 17-fold compared to cells before onset of starvation (Figure 2). As an additional control we tested the OASTL activity both in S-sufficient and S-starved cells at zero time (that is just washed in S-free medium prior to extraction), yielding perfectly comparable activity of the enzyme compared to S-sufficient growth 24 h later (data not shown). In other words, as observed earlier [15], OASTL activity did not change within 24 h in *Chlorella* at this growth stage under sufficient S supply.

According to [15] in *Chlorella sorokiniana* the synthesis of Cys is exclusively performed in the chloroplast under ample sulphur supply but induced in the cytosol in condition of S-deprivation. As shown in Figure 1 the increase of OASTL activity in S-starved cells can now be ascribed to the cytosolic isoform and to a large increase in the chloroplastic isoform.

## 3. Experimental Section

### 3.1. Algal Growth Condition and Sulphate Starvation

*Chlorella sorokiniana* Shihira & Krauss, strain 211/8 K (CCAP of Cambridge University) was grown under controlled conditions in batch culture (2 L) at 35° C, continuously illuminated (Philips TLD 30 W/55 fluorescent lamps, 250 µmol photons m^−2^·s^−1^), and flushed with air containing 5% CO_2_ at a flow rate of about 80–100 L·h^−1^. The basal medium had the following composition: 13 mM KH_2_PO_4_, 4.3 mM K_2_HPO_4_, 0.35 mM NaCl, 1.2 mM MgSO_4_, 0.35 μM Fe-EDTA, 0.18 mM CaCl_2_, 5 mM KNO_3_, oligoelements (0.31 μM Cu, 0.1 μM Mo, 9.1 μM Mn, 0.76 μM Zn, 46 μM B). The pH of the medium was adjusted to 6.5. Under these conditions, the cells (S-sufficient) growth rate constant (μ) was 3 d^−1^.

To induce S-deficiency, algal cells grown in the basal medium, were collected from batches during their exponential growth phase (culture OD between 0.5–1.0); the cells were harvested by a low speed centrifugation (4000× *g* for 5 min), washed two times and then re-suspended in the S-free medium containing 13 mM KH_2_PO_4_, 4.3 mM K_2_HPO_4_, 0.35 mM NaCl, 1 mM MgCl_2_, 0.35 μM Fe-EDTA, 0.18 mM CaCl_2_, 2.5 mM KNO_3_, and oligoelements (0.31 μM Cu, 0.1 μM Mo, 9.1 μM Mn, 0.76 μM Zn, 46 μM B). Cells were considered S-starved after 24 h of stay in the S-free medium.

### 3.2. OASTL Enzymatic Activities

*Chorella sorokiniana* cells from S-sufficient and S-starved (for 24 h) cultures (1 L) were harvested by low speed centrifugation (4000× *g* for 10 min). To the packed cells, 10 mL of extraction buffer (50 mM phosphate-buffer pH 7.5, 10 μM PLP and 1 mM dithiothreitol) were added. The cells were then broken by passing twice through a French pressure cell (1100 psi). The lysate was cleared by centrifugation at 15,000× *g* for 15 min at 4 °C and the obtained supernatant represented the crude extract. The enzymatic activity of OASTL was measured according to Gaitonde [33] method modified as described by Carfagna *et al.* [15]. One enzyme U is defined as 1 µmol cysteine min^−1^·mg^−1^ protein. The OASTL activity was related to the total soluble protein content of the samples; protein amounts were determined using the Bio-Rad protein assay based on the Bradford method [34] with bovine serum albumin as the standard.

### 3.3. OASTL Affinity Protein Purification

*Chlorella* crude extracts from S-sufficient and S-starved cultures were used for the purification of OASTL proteins by interaction with recombinant AtSAT5 (At5g56760). The crude extract preparation was already described in the previous paragraph (3.2).

AtSAT5 protein from *Arabidopsis thaliana* was over-expressed in *Escherichia coli* HMS174 (DE3) and immobilized on a nickel-loaded Hi-trap column (GE-Healthcare IMAC HP 0.7 × 2.5 cm^−1^ mL) as previously described [32]. The purification was carried out as described by Heeg and co-workers [32] and suitably modified for *Chlorella* cells.

*E. coli* cells expressing AtSAT5 (250 mL) were harvested and re-suspended with 10 mL of buffer containing: 50 mM Tris pH 8.0; 250 mM NaCl; 20 mM imidazole and 0.5 mM phenylmethanesulfonylfluoride. Bacterial cells were lysed by passing twice through a French pressure cell (1100 psi) and pelleted by centrifugation at 15,000× *g* for 15 min at 4 °C. The supernatant was the bacterial extract.

*E. coli* AtSAT5 crude extract was loaded onto the Hi-trap column previously nickel-loaded for at least 1 h. After a quick washing with the buffer W (50 mM Tris-HCl pH 8.0, 250 mM NaCl, 80 mM imidazole), bacterial OASTL was removed with elution buffer (50 mM Tris-HCl pH 8.0, 250 mM NaCl, 80 mM imidazole, 10 mM OAS). The column was then washed with buffer W.

*Chlorella* crude extract was passed through the column for at least 1 h and then OASTL proteins were eluted with 10 mL of buffer containing 0.5 M Tris pH 8.0, 250 mM NaCl and 50 mM OAS. The purified fractions containing the enzyme OASTL were analyzed by SDS-PAGE.

### 3.4. Silver Staining

Purified protein fractions from S-sufficient and S-starved cells were separated by SDS-PAGE 12% at 45 mA. Proteins loaded in each lane were 80 μg. After the electrophoretic run the purified OASTLs were visualized as brownish bands by silver staining according to [35].

## 4. Conclusions

In microalgae multiple OASTL protein isoforms have been characterized and the expression of their genes under different nutritional conditions analysed. From these findings they are believed to be equivalent to those described in *Arabidopsis*. The interaction between the algal OASTL and *Arabidopsis* SAT was shown by purification of OASTLs from *Chlorella sorokiniana*, suggesting for the first time, the existence of a SAT-OASTL interaction in algae like the CSC of vascular plants. The main aim of this study was to provide proof-of-concept for the suitability of a SAT-OASTL affinity purification system from land plants for the biochemical study of cysteine synthase complexes in the diverse taxa of algae. Furthermore, the specific activity of purified OASTLs in S-starved *Chlorella* cells was much higher compared to a similarly produced OASTL protein fraction from *Arabidopsis* leaf extracts.

OASTL was enriched 29-times from S-sufficient cells and 41-times from S-starved cells based on specific activity. The efficiency of this one-step purification strategy was very high with yields of 77% and 58%, respectively. This opens the possibility to also purify OASTL proteins from other algal taxa. Furthermore, this will allow us to obtain protein sequence information from species whose genome has not been sequenced, to determine biochemical properties of the enzyme and to assess the regulatory role of the cysteine synthase complex with respect to sulphate assimilation during nutritional and other environmental changes.
